# Draft Genome Sequences of *Citrobacter freundii* Strains CF04 and A41 Isolated from Moribund, Septicemic Giant Gourami (*Osphronemus goramy)* in Sri Lanka

**DOI:** 10.1128/genomeA.00820-16

**Published:** 2016-08-11

**Authors:** Karim Honein, S. S. S. De S. Jagoda, Appudurai Arulkanthan, Hideki Ushio, Shuichi Asakawa

**Affiliations:** a Laboratory of Marine Biochemistry, Department of Aquatic Bioscience, Graduate School of Agricultural and Life Sciences, The University of Tokyo, Yayoi, Bunkyo-ku, Tokyo, Japan; b Laboratory of Aquatic Molecular Biology and Biotechnology, Department of Aquatic Bioscience, Graduate School of Agricultural and Life Sciences, The University of Tokyo, Yayoi, Bunkyo-ku, Tokyo, Japan; c Center for Aquatic Animal Disease Diagnosis and Research, Department of Veterinary Pathobiology, Faculty of Veterinary Medicine and Animal Science, University of Peradeniya, Peradeniya, Sri Lanka

## Abstract

*Citrobacter freundii* is a Gram-negative opportunistic pathogen associated with many infectious conditions including septicemia in humans and animals. Here, we announce the draft genome sequences of two multidrug-resistant *C. freundii* strains (CF04 and A41) isolated from septicemic giant gourami (*Osphronemus goramy*) collected from aquaria in Sri Lanka.

## GENOME ANNOUNCEMENT

*Citrobacter freundii* is an infrequent but established cause of systemic infections in farmed food fish and aquarium fish (1, 2). During an investigation to identify septicemia causing bacterial pathogens of fresh water ornamental fish in Sri Lanka, the occurrence of *Citrobacter* spp. was only second to *Aeromonas* spp. (3). *C. freundii* has received increasing attention as an opportunistic pathogen that causes diarrhea, urinary tract infections, and septicemia in humans (4). However, to our knowledge the information on genome sequences of *C. freundii* isolated from diseased fish was not available. Further, *C. freundii* has shown a strong ability to developing resistance to antibiotics used in human therapy and aquaculture (5, 6). It is therefore imperative to advance our understanding of pathogenic, multidrug-resistant *C. freundii* strains associated with fish in order to identify antimicrobial resistance and virulence determinants.

Two *C. freundii* strains, CF04 and A41, were isolated from the kidneys of two moribund giant gourami (*Osphronemus goramy*) fish showing signs of generalized septicemia collected from aquaria in Kandy, Sri Lanka.

Genomic DNA was extracted with the DNeasy Blood and Tissue kit (Qiagen). Genomic DNA libraries were constructed using the Ion Xpress Plus fragment library kit and sequenced on an Ion Torrent PGM (Life Technologies) platform using the ion 318 chip and the 400-bp kit following standard protocols. A total of 1,328,195 reads equivalent to 340,760,015 bp of data and 1,170,722 reads equivalent to 330,995,850 bp of data were, respectively, generated from *C. freundii* CF04 and A41 DNA samples. The reads acquired were assembled separately using MIRA version 4.0.2 (7) and improved with CLC Genomics Workbench version 8 by alignment against a reference genome (accession no. CP007557). The qualities of the assemblies were evaluated using QUAST version 3.0 (8).

The assembly of the *C. freundii* CF04 strain resulted in 58 contigs over 1,000 bp (largest contig 1,274,500 bp; *N*_50_ = 679,933 bp) with a total length of 5,138,911 bp and 51.49% G+C content. The reads of *C. freundii* A41 were assembled to 153 contigs larger than 1,000 bp (largest contig 548,564 bp; *N*_50_ = 366,314 bp) with a total length of 5,301,254 bp and 51.5% G+C content. The total size of the draft genomes and G+C contents are in agreement with published *C. freundii* genomes (4.9 to 5.0 Mb and 51.6% G+C) (9–11).

Functional annotation using RAST (12) revealed 5,777 and 5,253 coding sequences (CDS) in the draft genomes of CF04 and A41, respectively. The presence of genes involved with virulence, disease and defense, quorum sensing, and biofilm formation as well as genes encoding resistance to different antibiotics confirms the clinical significance of these isolates. Moreover, RAST indicated that both organisms carry genes encoding resistance to antibiotics that include fluoroquinolon, macrolides, beta-lactams, rifampin, chloramphenicol, tetracycline, aminocoumarin, aminoglycoside, polymyxin, and vancomycin. PHAST, a phage search tool (13), predicted six intact phages in CF04 and seven intact phages in A41.

### Accession number(s).

The draft genome sequences of *C. freundii* A41 and CF04 have been deposited in DDBJ/EMBL/GenBank under the accession numbers BDFK01000001 to BDFK01000153 and BDFL01000001 to BDFL01000058, respectively.
